# Respiratory biofeedback and psycho-education for patients with post COVID- 19 symptoms and bodily distress: study protocol of the randomized, controlled explorative intervention trial POSITIV

**DOI:** 10.1186/s13063-025-08842-6

**Published:** 2025-04-25

**Authors:** Hannah Dinse, Eva-Maria Skoda, Adam Schweda, Christoph Jansen, Kira Schmidt, Margarethe Konik, Hana Rohn, Oliver Witzke, Mark Stettner, Christoph Kleinschnitz, Alexander Bäuerle, Martin Teufel

**Affiliations:** 1 Clinic for Psychosomatic Medicine and Psychotherapy, LVR-University Hospital, University of Duisburg-Essen, Virchowstraße 174, Essen, 45147 Germany; 2 Department of Infectious Diseases, West German Centre of Infectious Diseases, University Hospital Essen, University of Duisburg-Essen, Hufelandstrasse 55, Essen, 45147 Germany; 3 Department of Neurology, University Medicine Essen, University of Duisburg-Essen, Hufelandstrasse 55, Essen, 45147 Germany; 4 Center for Translational Neuro- and Behavioral Sciences (C-TNBS), University of Duisburg-Essen, Hufelandstrasse 55, Essen, 45147 Germany

**Keywords:** Long COVID- 19, SARS-CoV- 2, Bodily distress disorder, Somatic symptom disorder, Randomized controlled trial, Biofeedback, Cognitive behavioral therapy, Psychosomatic, Psychotherapy

## Abstract

**Background:**

A high number of individuals report suffering from physical and psychological sequelae symptoms after COVID-19—the so-called post COVID-19 condition. Commonly reported complaints include physical symptoms such as fatigue, headache, attention and concentration deficits or dyspnea and anxiety, symptoms of post-traumatic stress, or depression. Evidence-based treatment recommendations are still lacking up to this point. Associations between physical and psychological symptoms in chronic diseases are known for a long time. Support in coping with the disease and improvement of self-efficacy can have a positive effect on the course of diseases. For this reason, we designed a randomized, controlled explorative intervention trial as a treatment of bodily distress disorder in COVID-19 recovered persons.

**Methods:**

Patients with a post COVID-19 condition meeting to the criteria of the WHO, along with a bodily distress disorder, are randomized in an intervention and control arm (TAU). Randomization takes place after a diagnostic interview, screening, and informed consent. In total, 60 patients will be included in the trial (30 per group). The intervention group receives a cognitive behavioral therapy as a video-conference-based group therapy (6 weeks) and mobile, respiratory biofeedback treatment (for 4 weeks). At several time points, both groups are assessed in terms of psychological and physical health status, treatment expectation, and satisfaction with the intervention. Furthermore, they will get biofeedback examination appointments. The primary outcome is the change in self-efficacy; secondary outcomes include assessed parameters of mental health, somatic symptoms, and satisfaction with the intervention. Data will be analyzed primarily using R and SPSS.

**Discussion:**

The randomized, controlled, explorative intervention trial POSITIV is one of the very first interventions for patients with post COVID-19 condition and psychological burden due to their different symptoms. The aim of the study is to generate new evidence and help patients to cope with the disease and thus, increase their quality of life and reduce symptomatology. We expect with a high probability that the patients’ self-efficacy and health status will improve as a result of the intervention.

**Trial registration:**

German Clinical Trial Register (DRKS); DRKS-ID: DRKS00030565. Registered on December 22, 2022.

**Supplementary Information:**

The online version contains supplementary material available at 10.1186/s13063-025-08842-6.

## Background

Many people who recovered from COVID- 19 described somatic and psychological sequelae symptoms weeks and months after an acute COVID- 19 infection [1]. Current data estimate that 10% of all who were infected suffer from such sequelae symptoms [2]. Due to the heterogeneity and novelty of the disease, there was no clear medical definition for prolonged COVID- 19 symptoms for a long time [3]. In late 2021, the World Health Organization (WHO 2022) coined the term “post COVID- 19 condition” [4]. It is defined as a disease or impairment that occurs in persons who have experienced a SARS-CoV- 2 infection, and which results in symptoms usually occurring within 3 months after COVID- 19. Such symptoms are expected to persist for at least 2 months and must not be explainable by any other diagnosis [4].

In clinical practice, the post COVID- 19 condition presents as a heterogeneous syndrome. According to systematic reviews, the five most common symptoms are fatigue (58%), headache (44%), attention deficit symptoms (27%), hair loss (25%), and dyspnea (24%) [5, 6]. Insomnia, anxiety, symptoms of post-traumatic stress disorder, depression, and somatization have been reported as the most common psychological symptoms.

Already at early stages of the pandemic, it became apparent that COVID- 19 patients experience psychological distress both during and after the acute phase of illness [6–11]. Multiple factors were discussed to be relevant. Among others, authors have mentioned insecurities, fears about the consequences of the infection, stigmatization, concern about having infected another person, social isolation, and/or the stressful and unfamiliar experience or situation during inpatient (intensive care) hospitalization [12–14]. Patients often report psychological burden due to the prominent somatic symptoms, which are, in turn, accompanied by concerns about the disease progress and its impact on their daily life.

In fact, chronic somatic diseases often cause psychological distress. Typical diseases that potentially cause massive psychological distress include oncological, but also neurological and cardiological illnesses.

The treatment and diagnostic guidelines for such oftentimes chronic diseases already suggest psychological treatment or care for patients who wish to receive support [15, 16]. Moreover, the German guideline for post COVID- 19 treatment explicitly mentions psychosomatic treatment options [17].

Psychological burden due to an underlying somatic disease may lead to a bodily distress disorder also called somatic symptom disorder, as defined in the DSM- 5 as well as ICD- 11 [18–20]. Due to the versatility of symptoms on both, the physiological and psychological level, an interdisciplinary treatment concept is necessary. Treatment should be symptom- and patient-oriented. A staged concept depending on the severity and complexity of the course is preferable.

At the moment, there is no science-based knowledge on psychological treatment of the patients with a post COVID- 19 condition with psychological burden. However, intervention studies for bodily distress disorders—mostly in combination with somatic diseases—show the most promising results up to this point when including cognitive behavioral therapy (CBT)-based methods, as well as therapies that include the direct regulation of the stress, arousal, and bodily sensations [21, 22].

With this knowledge, we have designed our randomized controlled study, which focuses on videoconference-based CBT-based group intervention and a mobile respiratory biofeedback treatment. This integrative intervention will focus on strengthening patient’s self-efficacy. The aim of this RCT study is to generate new evidence in the treatment of patients with post COVID- 19 specifically targeting the psychological burden that goes along with it. We intend to help patients to cope with the disease and the accompanying repercussions via CBT-based and biofeedback-based interventions. Here, we use self-efficacy experience as a primary outcome measure. In the second place, we will assess the patient’s mental health and somatic symptoms as well as intervention’s acceptability by measuring treatment satisfaction, expectation, feasibility, and acceptance directly.

## Methods/design

### Study design

The study is an exploratory randomized controlled trial with two parallel study arms. After screening and recruitment, participants will be randomized into the two study groups. Participation is voluntary. The recruitment itself started in October 2022.

The study protocol is based on the SPIRIT guidelines [23]. The checklist can be found in the appendix.

**Fig. 1:**
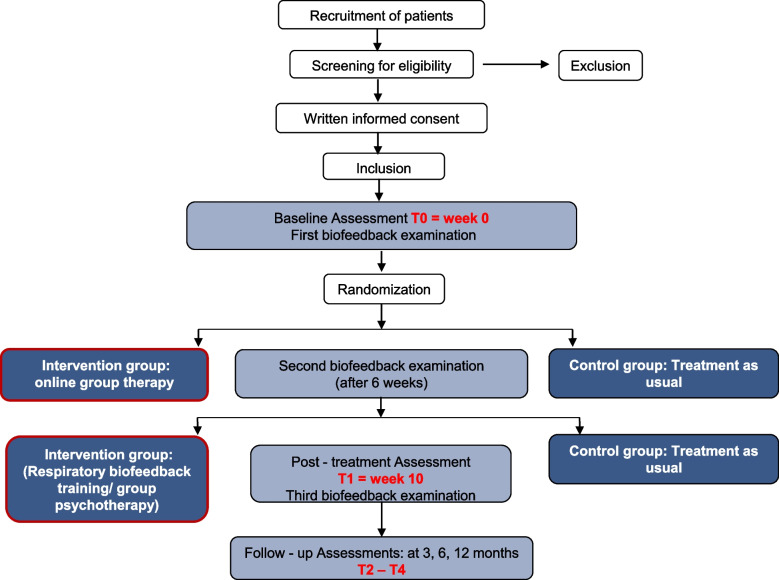
Flow chart of the POSITIV trial

Participants receive pre-treatment assessments at baseline (T0) and post-treatment (T1), as well as at three follow-up assessments (3, 6, 12 months after the intervention; T2–T4). Patient data is collected and stored in strictly pseudonymous form. Participants can decide to refrain from participation at any time. In this case, participants will be asked to complete an assessment regarding reasons, expectations, and changes (see Fig. 1).

### Participant recruitment

Recruitment takes place via outpatient clinics (usually general physicians and post COVID- 19 specialists), newspaper articles, social media, self-help groups, by telephone or e-mail, as well as by mail. The Clinic for Psychosomatic Medicine and Psychotherapy (LVR-University Hospital) with support of the Department of Infectious Diseases (University Hospital Essen) was responsible for recruitment. The recruitment started in October 2022 and is scheduled to be completed in September 2023.

### Inclusion criteria

We include adult participants (18 years or older) with sufficient knowledge of German language to allow active participation in psychological interventions. Interested participants must meet the diagnostic criteria for bodily distress disorder (see ICD- 11 or DSM- 5 criteria) [19, 20] and the criteria for a post COVID- 19 condition (WHO Delphi Consensus diagnostic criteria) [4]. Bodily distress disorder (ICD- 11) involves excessive focus on distressing physical symptoms, accompanied by disproportionate worry or health-related behaviors. Symptoms persist for several months, significantly impairing daily functioning [24]. Furthermore, symptom severity does not exceed a critical level so that an inpatient treatment is necessary. Participants must give written informed consent.

Inclusion interviews are always conducted in the outpatient clinic for Psychosomatic Medicine and Psychotherapy (LVR-University Hospital Essen) by the same female physician who also conducts the experimental interventions (videoconference-based cognitive behavioral group intervention). Patients should not participate another bio- or neurofeedback intervention during the study.

### Intervention: mobile biofeedback treatment and videoconference-based cognitive behavioral group intervention

The first part of the intervention includes element of cognitive behavioral therapy. The therapy is conducted online as a group therapy with about four up to seven participants. It takes place weekly for 6 weeks. One therapy session lasts 60 min. In the six modules, the participants focus on the following topics: stress/distress, resources, emotions, behavior, thoughts and recognizing own needs and limits, future perspectives and goals. The videoconference systems (*samedi GmbH*; Berlin, Germany or *RED Medical* Systems GmbH; Muenchen, Germany) allow patients to participate in therapy from home. Both digital providers are approved by health insurances for online psychotherapy.

The second part of the intervention is a mobile respiratory biofeedback treatment. The focus on respiration was chosen because of the frequently named symptom of dyspnea in post COVID- 19 patients. The respiratory biofeedback treatment is performed in the patients’ home environment (4 weeks), and the behavioral cognitive therapy takes place as a videoconference-based group intervention (6 weeks). The mobile biofeedback devices (*eSense Respiration*) are a *Mindfield® Biosystems Ltd.* (Hindenburgring 4; Gronau, Germany) product. The product includes a breathing sensor—which monitors breathing frequency, amplitude, and its pattern in connection with *eSense skin response*. A mobile application makes it possible to perform the biofeedback training at home, although support by the provider will be offered. Before starting the respiratory biofeedback treatment, patients receive a detailed technical education with an additional written instruction.

The feedback itself might be visualized in different tasks, such as bar feedback, curve feedback, video feedback, music feedback, tone feedback, tactile feedback through vibration, or free training. Patients are recommended to exercise at least twice a week for 15 min each time. Changes and successes are recorded by the app and can be evaluated later.

### Control intervention: TAU

A TAU-based control group was selected as control group. Patients will have access to standard healthcare during the trial. The treatment as usual (TAU) includes all currently existing measures of support and treatment available to patients in somatic and psychosomatic care (e.g., outpatient individual or group psychotherapy, (partial) inpatient psychosomatic treatment, nutritional counseling, social work, physical therapy. self-help group, online interventions).

### Outcomes

The primary aim is to evaluate an intervention for the treatment of bodily distress disorder in COVID- 19 recovered persons. This will address an existing gap in the medical care system in Germany and provide new evidence in the treatment of a bodily distress disorder going along with a post COVID- 19 condition.

#### Primary outcome measure

The primary outcome is the change of self-efficacy at the end of the treatment (T1). To assess the self-efficacy of the participants, the 10-item *General Self-Efficacy Scale* [GSES] is used [25]. The scale was created to capture of perceived self-efficacy which reflects an optimistic self-belief. This can be a positive resource factor for resistance in accomplish new or difficult tasks or cope with new burden. We used the German version of the assessment [25].

#### Secondary outcomes measure

The secondary outcomes are used to monitor of assessed parameters on mental health, somatic symptoms, and quality control of the intervention. This includes (1) distress, (2) depression, (3) anxiety, (4) quality of life, (5) symptoms of a post-traumatic stress disorder, (6) sense of coherence, (7) somatic symptom burden, (8) symptoms of somatic symptom disorder, (9) symptoms of post COVID- 19, (10) patient satisfaction, (11) patient motivation, and (12) patient expectation (Table 1).

**Table 1:** Overview of contents of the assessment at different points in time

Measures	Baseline T0	End of treatment T1	Follow-up assessments T2–T4	Dropout assessment
**Primary outcome measure**				
General Self-Efficacy Expectancy Scale [GSES]	x	x	x	
**Secondary outcomes measure**				
Distress Thermometer	x	x	x	x
The Patient Health Questionnaire Depression Scale [PHQ- 8]	x	x	x	x
Generalized Anxiety Disorder Scale- 7 [GAD- 7]	x	x	x	x
The World Health Organization Quality Of Life Questionnaire [WHOQOL-BREF]	x	x	x	x
The Revised Impact of Event Scale [IES-R]	x	x	x	
Sense of Coherence Scale—Leipzig Short Form [SOC-L9]	x			
Somatic Symptom Scale- 8 [SSS- 8]	x	x	x	x
The Somatic Symptom Disorder—B Criteria Scale [SSD- 12]	x	x	x	x
The course and symptoms of the COVID- 19 infection	x			
Post COVID Syndrome [PCS] Score	x	x	x	x
**Measures exclusively for intervention group**				
Client Satisfaction Questionnaire [CSQ]		x		x
Motivation regarding the intervention	x	x	x	
Expectations regarding the intervention	x	x	x	
Engagement with the exercises		x	x	x
Reasons for dropout				x

The variables are as followed:

1. Distress: The *Distress Thermometer* is an 11-point visual analog scale which inquires the patients to indicate their distress over the past week (including today). The scale ranges from 0 (no distress) to 10 (worst-possible distress) [26].
2. Depression: The *Patient Health Questionnaire Depression Scale* [PHQ- 8] has eight items and is designed to diagnose and determine the severity of depression [27].
3. Anxiety: This is measured with the *Generalized Anxiety Disorder Scale- 7* [GAD- 7]. The GAD- 7 is a seven-item assessment used to evaluate generalized anxiety disorder [28].
4. Quality of life: The *World Health Organization Quality Of Life Questionnaire* [WHOQOL-BREF] assesses quality of life [29]. It was developed in 1998 as a shortened version of the WHO-QOL- 100 and includes four domains related to quality of life: physical health, mental health, social relationships, and environment.
5. Symptoms of a post-traumatic stress disorder: The *Revised Impact of Event Scale* [IES-R] captures symptoms and typical reactions to stressful events such as natural disasters or serious accidents on three subscales: intrusions, avoidance, and overexcitement [30]. The IES-R is well suited for measuring symptom severity and has satisfactory psychometric characteristics. In our study, the questions in the IES-R regarded the COVID- 19 infection.
6. Sense of coherence: This is considered a dispositional coping resource that makes people more resilient to stressors. The *Sense of Coherence Scale—Leipzig Short Form* [SOC-L9] is used to measure the sense of coherence of patients and subjects in the sense of Antonovsky [31]. It is a one-dimensional scale consisting of 9 items.
7. Somatic symptom burden: The burden due to somatic symptoms is measured by the *Somatic Symptom Scale- 8* [SSS- 8] [32]. It is a validated patient-reported outcome assessment.
8. Symptoms of somatic symptom disorder: The *Somatic Symptom Disorder—B Criteria Scale* [SSD- 12] is an instrument that operationalizes the psychological characteristics of a Diagnostic and Statistical Manual of Mental Disorders, Fifth Edition somatic symptom disorder [33]. It consists of 12 items.
9. Symptoms of post COVID- 19: To assess the post COVID- 19 symptoms of the participants, we used the *Post COVID Syndrome (PCS) Score* [34].
10. Patient satisfaction: The *Client Satisfaction Questionnaire* [CSQ] is a screening assessment consisting of 8 items that measures patient satisfaction [35].
11. Patient motivation: We asked patients to estimate their motivation regarding the biofeedback treatment and group intervention on a scale from 0 to 100 (none, very high). Moreover, participants were asked to indicate, how often they performed the exercises presented the group intervention or biofeedback treatment (0 = almost never; 5 = almost daily).
12. Patient expectation: Furthermore, the patients were asked about their treatment-related expectations. These questions were scaled from 0 to 100 (none, very high). In an open question, respondents were asked about content in group therapy that they particularly liked or disliked.

### Biofeedback examination

Biofeedback assessment will take place at measurement before the group intervention, between the group intervention and the biofeedback training, and after the biofeedback training (week 0, 6, and 10). Different physiological measures will be assessed using the Nexus- 10 device (MindMedia, Herten, Germany): respiratory rate, blood volume pulse, shoulder–neck electromyography, and electroencephalography. The diagnostic assessment follows a 14-min protocol. Participants will undergo a 2-min baseline measurement, 2-min stress induction by using the Stroop test [36, 37], 2-min relaxation (beach images and calm music), 2-min stress induction by mental arithmetic (based on Kirschbaum et al.) [37], 2-min relaxation (beach images and calm music), 2-min stress induction by reporting a recent stressful event, and 2-min relaxation (beach images and calm music [36, 37]).

### Sample size calculation

The power analysis was performed for a 2 (between-subjects, treatment vs. control) × 5 (within-subject, measurements T0–T4) design where the primary focus was the interaction effect between the treatment and the time variable. We used G*Power [38]. In order to be able to find an effect of *η*_*p*_^2^ = 0.03 with a test power of 1 − *β* = 0.80, which falls between what is considered a small and medium effect size [39], a total sample of 44 participants is needed.

Based on Cooper and Conklin, we assume a non-negligible dropout rate [40]. Therefore, we planned to assign 30 participants to both the treatment and control conditions, summing to a total sample of *n* = 60.

### Data management, data storage, and dissemination

The data are collected in a pseudonymous fashion and will be stored for 10 years (in conformance with the Good Clinical Practice guidelines [25]). The study will be subject to European Data Protection Regulation (EU-DSGVO). In detail, patient records will be kept in accordance with hospital policy, and access to stored data will be restricted to authorized personnel. We will publish key study results in an open-access, peer-reviewed journal and make publicly available via the Clinical Trials Registry. In addition, we will present the results at conferences and communicate the scientific results in plain language through press releases, social media, or patient forums.

The Project Management Group will meet weekly to review the trial conduct.

After the findings of the study are published, we want to make the collected data available in anonymized form upon reasonable request. We will retain the statistical analysis plan and relevant documents and make them available upon request. Overall, the patient consent forms include a section addressing the above aspects of data storage and sharing.

### Randomization and blinding

For randomization, a standard computer algorithm is used with the function of a 1:1 randomization. Randomization is carried out by the person conducting the inclusion interview. Due to the study design, a complete blinding for patients will be impossible.

However, the final analysis is performed by a blind analyst to ensure objective evaluation.

### Statistical methods

The aim of the study is to analyze the efficacy of the intervention in terms of improvement of self-efficacy as well as mental and physical health. This will take place in comparison between the intervention and control group. In the evaluation, we will perform an analysis of covariance (ANCOVA) with the outcome measured at T1 and the baseline scores of T0.

First of all, the analyses will be performed according to the intention-to-treat principle. Missing data will be imputed. Therefore, SPSS multiple imputation, module employing “monotone missing pattern” and incorporating complete data for sex, age, and baseline measurements of primary and secondary outcomes are used. We do not plan interim analyses.

Secondly, to evaluate the sustainability of the effects in comparison of both groups (between-subject factor) and of the different measurement points (T0–T4; with-subject factor) mixed ANOVAs will be conducted. In case of massive violations of normality, we will resort to robust methods such as generalized estimating equations. In case of violation of assumption of sphericity, we will use Huynh–Feldt or Greenhouse–Geisser corrections. Further descriptive analyses will assess treatment motivation, expectations, satisfaction, clinical data, and sociodemographic data. To illustrate potential group differences, *t*-tests and χ^2^ tests will be used. In case of non-normality or small cell sizes, the Mann–Whitney *U* tests or Fisher’s exact tests will be used. Further potential exploratory analyses might include subgroup comparisons, such as sex differences, dropouts, or participants who have the most benefit. Third, we would like to explore possible correlations and association patterns using regression analysis or network analysis. The documentation of adverse events and serious adverse events is conducted in separate tables and line listings for comprehensive analysis to ensure the highest level of safety, although we do not anticipate any serious or adverse events. However, should any unexpected serious adverse events occur (such as suicidal risks or severe depressive symptoms), participants will have access to expert consultation from the study team to ensure they receive the necessary support.

### Ethical aspects

The study is conducted in accordance to the guidelines of the Declaration of Helsinki and approved by the local Ethics Committees of the University Hospital Essen (22–10,844-BO). In the event of important protocol modifications, the study center will immediately inform the ethics committee and, if necessary, the sponsor and participants. In addition, we will update the protocol in the clinical trial registry.

### Roles and responsibilities

The clinical trial oversight involves several key groups. The coordinating center, steering committee, and data management team take place through the Clinic for Psychosomatic Medicine and Psychotherapy, LVR-University Hospital. The coordinating center manages overall operations, communication, and quality control, while the steering committee provides strategic guidance and ensures scientific and ethical oversight. The data management team focus on unbiased endpoint evaluation and data quality, ensuring the trial meets regulatory and scientific standards. Additional teams are the Ethics Committees of the University Hospital Essen, which ensure the trial approval and ongoing ethical compliance.

## Discussion

The POSITIV trial is, to our knowledge, the first randomized controlled trials for patients with post COVID- 19 and psychological burden using respiratory biofeedback training and cognitive behavioral group therapy as intervention.

What is unique about the presented intervention is that it is based on the consideration of the multifactorial pathogenesis of post COVID- 19 [41–46]. For this reason, patients in the intervention group will receive respiratory biofeedback training, which can influence by learning processes the autonomic nervous system, in addition to cognitive behavioral group therapy to support coping with the disease. Thus, the aim is to improve patients’ self-efficacy in comparison to the control group.

Since COVID- 19 spreads worldwide, the number of people with sequelae symptoms after infection increases. As somatic symptoms, impairments of the autonomic nervous system (ANS) appear to be common in post COVID- 19 patients [47, 48]. An ANS dysfunction can course changes in the regulating of physiological functions as respiration, blood pressure, heart rate, or digestion [48].

The main central nervous regulator of the ANS is the hypothalamus with its nuclei as well as the brainstem with the regions of midbrain, pons, and medulla oblongata [49, 50]. The exact pathomechanisms of ANS dysfunction after COVID- 19 are still speculative. Different studies in the last year focused on potential mechanism behind autonomic dysfunction of post COVID- 19, identifying neuroinflammation, autoimmunity, and disruption in the renin-angiotensin system or endothelial damages as key contributors [48, 51, 52]. Dani et al. hypothesize that the autonomic nervous system may be disrupted by viral or immune-mediated mechanisms, leading to either transient or persistent orthostatic intolerance syndromes [51]. In the therapy of ANS dysregulation, biofeedback methods are used for self-regulation. Studies show a significant improvement in biofeedback training for stress-related disorders such as post-traumatic stress disorder, depression, or panic disorders [53, 54]. In addition, an efficacy in combination with cognitive behavioral therapy could be shown. Changes were visible after 4 weeks of training [53]. Due to the altered respiratory regulation caused by the impairment of the ANS, and based on the previous research results, we integrated the respiratory mobile biofeedback treatment as a part of the study intervention.

Many post COVID- 19 symptoms cause difficulties in individuals’ functioning levels. Furthermore, this often leads to additional psychological burden due to worries, anxiety, and fears about the course of the disease [55, 56].

The German guidelines for treatment of post and long COVID- 19 recommended to consider diseases and dysfunctional coping strategies in the treatment of post COVID- 19 psychological symptoms [17, 57]. Prior studies on rehabilitation treatment of post COVID- 19 patients showed significant benefits of including individual and group psychotherapy (CBT), individualized aerobic exercise training, body awareness training, breathing therapy, relaxation techniques, cognitive training, and social counseling as core elements [45, 58]. A Dutch RCT study investigating the effect of CBT on post COVID patients with fatigue demonstrated that participants reported improvements in physical resilience and social life [59, 60]. The positive effects of the therapy persisted for up to 1 year after the intervention, highlighting the sustainability of CBT in treating long COVID symptoms [61].

Studies also showed that post COVID- 19 patients have a clinically significant psychological burden, and that such treatment concept could help to reduce this kind of distress [45, 58], which is why we decided to develop and assess further psychological treatment possibilities.

In the execution of this RCT, there are challenges to consider. First, post COVID- 19 patients exhibit a very heterogeneous symptomatology both somatically and psychologically. Yet, a concentration deficit as well as a fatigue complex appears to be very central and quite common [62]. This can result in difficulties in a proper utilization of treatment options due to, e.g., restrained ability to travel. Second, a requirement is the technical equipment for participation in the group therapies, which constitutes a further limiting factor.

In conclusion, this trial might improve our knowledge about a treatment of bodily distress disorder in COVID- 19 recovered persons. This knowledge could be helpful in the health care of patients with the post COVID- 19 condition.

## Trial status

The trial is registered in the German Clinical Trial Register (DRKS; DRKS-ID: DRKS00030565). This study protocol is the first version for this specific trial. The recruitment started on October 5 th of 2022. Approximately the recruitment is completed in September 2023. Recruitment is complete when the paper is submitted. This was due to the clinic’s urgent care for post COVID- 19 patients. It was necessary to provide prompt and rapid care to the burdened patients, who could not wait for a publication to be completed. End of September 2024, the complete follow-up will be finished.

## Supplementary Information

Additional file 1. SPIRIT figure

Additional file 2. SPIRIT checklist

## Data Availability

Study data will be published and made available to the scientific community after the assessments are completed.

## Abbreviations

ANS: Autonomic nervous system
CBT: Cognitive behavioral therapy
C-TNBS: Center for Translational Neuro- and Behavioral Sciences
CSQ: Client Satisfaction Questionnaire
DRKS: “Deutsches Register Klinischer Studien” (German Clinical Trials Register)
DSM- 5: Diagnostic and Statistical Manual of Mental Disorders
e.g.: Exempli gratia, for example
EU-DSGVO: “EU-Datenschutz-Grundverordnung” (EU General Data Protection Regulation)
GAD- 7: Generalized Anxiety Disorder Scale-7
GSES: General Self-Efficacy Expectancy Scale
IES-R: Impact of Event Scale—Revised
PCS-Scale: Post COVID Syndrome—Scale
PHQ- 8: Patient Health Questionnaire Depression Scale
RCT study: Randomized controlled trial study
SOC-L9: Sense of Coherence Scale—Leipzig Short Form
SSD- 12: The Somatic Symptom Disorder—B Criteria Scale
SSS- 8: Somatic Symptom Scale-8
TAU: Treatment as usual
WHO: World Health Organization
WHOQOL-BREF: World Health Organization Quality of Life-BREF
