# Supplementary material for: Respiratory biofeedback and psycho-education for patients with post COVID- 19 symptoms and bodily distress: study protocol of the randomized, controlled explorative intervention trial POSITIV
Source: Trials. 2025 Apr 25;26:140. doi: 10.1186/s13063-025-08842-6 (PMC12032681; doi:10.1186/s13063-025-08842-6)
Supplement: Supplementary file 1 — Additional file 1. SPIRIT figure [file 13063_2025_8842_MOESM1_ESM.pdf]

	Study Period					
	Enrollment	Allocation	Post-allocation			
**TIMEPOINT**	-t1	t0	t1	t2	t3	t4
**ENROLLMENT:**						
Eligibility screen	x					
Informed consent	x					
Allocation		x				
**INTERVENTIONS:**						
Intervention group		x	←	—	—	→
Control group		x	←	—	—	→
**ASSESSMENTS:**						
baseline variables	x	x				
outcome variables		x	x	x	x	x
